# Unusual presentation of a first Branchial cleft cyst associated with an abnormal bony canal -a case report-

**DOI:** 10.1186/s40463-020-00426-5

**Published:** 2020-05-29

**Authors:** A. Fanous, V. Couloigner, P. Gorphe, L. Galmiche, M. Alexandru, E-N Garabedian, L. Coffinet, T. Blanc, N. Leboulanger, F. Denoyelle

**Affiliations:** 1 Department of Otolaryngology - Head and Neck Surgery, McGill University, Quebec, Canada; 2 Department of Pediatric Otolaryngology – Head and Neck Surgery, Hôpital Necker – Enfants Malades – Paris V University, Paris, France; 3 Department of Otolaryngology – Head and Neck Surgery, Institut Gustave Roussy, Villejuif, France; 4 Pathology Department, Hôpital Necker – Enfants Malades – Paris V University, Paris, France; 5 Department of Otolaryngology – Head and Neck Surgery, Centre Hospitalier Régional et Universitaire de Nancy, Hôpital Central, Nancy, France; 6 Department of Pediatric Surgery, Hôpital Necker – Enfants Malades – Paris V University, Paris, France

**Keywords:** First branchial, Cholesteatoma, Robotic, Cyst, Parapharyngeal

## Abstract

**Background:**

First branchial cleft anomalies are rare, accounting for only 10% of all branchial cleft anomalies. We report an even more rare and unique case of a branchial cleft cyst with features of both first and second arch derivatives.

**Case presentation:**

A 6-year-old boy presented to us with a left conductive hearing loss associated with pre-tympanic keratin debris and an ipsilateral painful cervical mass. He had a past medical history of left ear surgery for presumed cholesteatoma 2 years prior and left neck abscess drainage 6 months prior. CT and MRI revealed a lesion originating in the external auditory canal and extending cervically through a bony canal located medial to the facial nerve and terminating as a parapharyngeal cyst. The complete removal was accomplished in one surgical stage consisting of three distinct steps: robotic assisted transoral resection of the pharyngeal cyst, an endaural approach and a parotidectomy approach.

**Conclusion:**

We believe that our detailed description of this rare first branchial cleft cyst with pharyngeal extension, possibly a hybrid case between a first and second branchial cyst, can serve as a valuable tool to Otolaryngologists – Head and Neck Surgeons who come across a similar unusual presentations.

## Background

Branchial cleft anomalies are the second most common congenital neck masses in the pediatric population, following thyroglossal duct cysts [1]. However, within the diagnosis of branchial cleft anomalies, first branchial cleft cysts are a rare occurrence, accounting for less than 10% of cases. In 1972, Work et al. proposed a classification with two distinct types of first branchial cleft cysts [2]. Type I cysts are superficial to the facial nerve and lie in close proximity to the ear, while type II cysts lie medial to the facial nerve and communicate with the external auditory canal. We will describe in this article an exceptional case of a first branchial cleft cyst presenting as a presumed ear canal cholesteatoma in association with an abnormal bony canal and a pharyngeal cyst.

## Case presentation

### Case background

A 6-year-old boy was referred to our pediatric Otolaryngology – Head and Neck Surgery department in March 2017 for presumed persistent left cholesteatoma. He had undergone surgery in 2015 at an outside institution. According to the operative report, keratin debris was encountered at the anterior-inferior portion of the external auditory canal in close proximity to the tympanic membrane. Faced with an uncertain diagnosis, the decision was made to abort the resection and refer the patient to a tertiary care institution. The post-operative audiogram (performed 6 months later) revealed normal right hearing and a mild left conductive hearing loss. In September 2016, he presented to the emergency department with a left sided painful cervical mass. CT scan revealed a lateral cervical abscess with para-pharyngeal extension. The abscess was drained shortly thereafter in the operating room, by both cervical and oral routes. The boy is otherwise healthy with no significant past medical history.

**Fig. 1:**
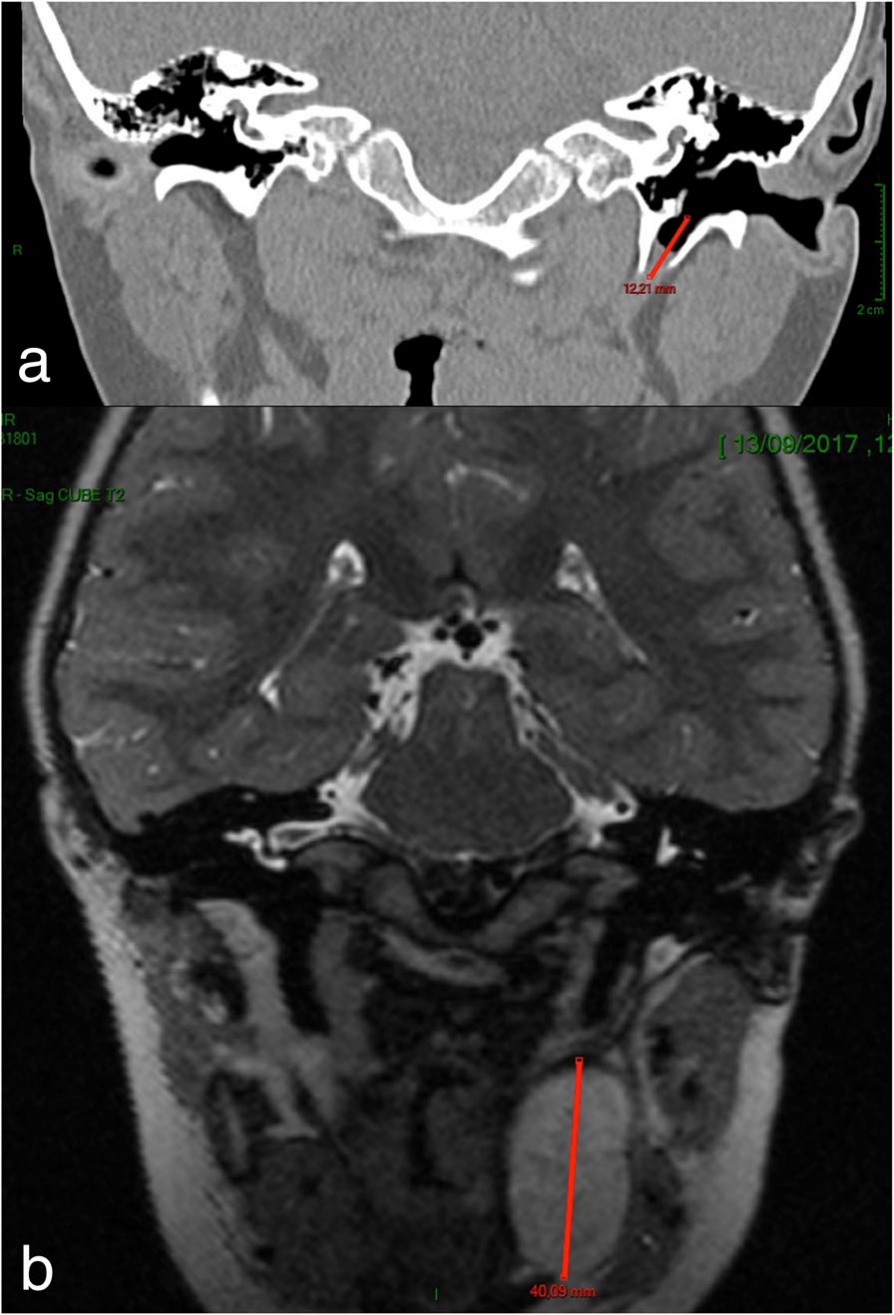
Imaging. **a**. Coronal CT scan and **b**. Coronal T2 weighted MRI key images demonstrating the bony canal lying medial to the facial nerve and a voluminous pharyngeal cyst

Physical examination at the time of initial consultation at our institution revealed normal tympanic membranes bilaterally. However, on the left side, a voluminous pre-tympanic cavity filled with ceruminous debris was observed. Computed tomography examination (CT scan) of the neck and temporal bones revealed a normal left middle ear with the presence of a bony canal in the deep portion of the tympanic bone originating from the pre-tympanic region. The canal lied in close proximity to the styloid process, which was eroded in its inferior portion, and was situated in between the carotid artery and the jugular vein (Fig. 1a). Magnetic resonance imaging (MRI) revealed a large left peri-tonsillar cyst measuring 3 × 1.7 cm, with regular and well-defined borders. Interestingly, there was no obvious cervical communication tract between the cyst and bony canal (Fig. 1b).

### Description of the surgical intervention

The surgery was performed in a single stage divided into three distinct parts: trans-oral robotic assisted parapharyngeal resection, an endaural approach (canaloplasty) and a parotidectomy approach. The surgery was performed under NIM facial nerve monitoring.

#### Trans-oral robotic assisted parapharyngeal resection

**Fig. 2:**
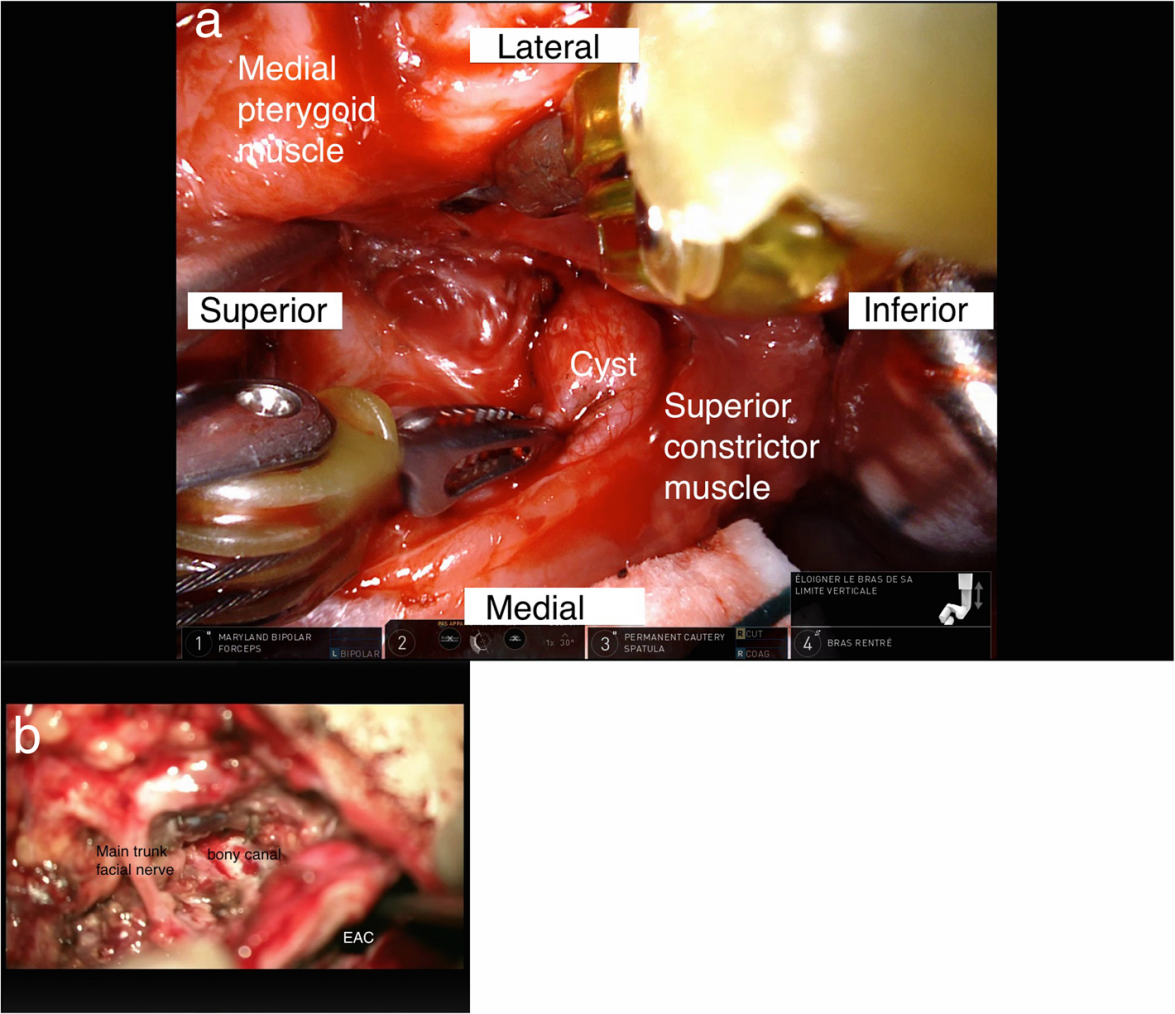
Intra-operative photographs. **a**. Robotic-assisted photograph of the pharyngeal cyst excision. **b**. Microscope image of the bony fistulous tract. The facial nerve and the external auditory canal (EAC) are labeled

This step was robot assisted (Intuitive Surgical - da Vinci Surgical System Xi®, Sunnyvale, CA, USA). Careful dissection of the voluminous parapharyngeal cyst was carried out using a combination of monopolar cautery, Harmonice ACE® curved shears (Ethicon Inc., Somervielle, USA), Maryland® bipolar forceps (Intuitive Surgical Inc., Sunnyvale, USA) and microscissors. The deep surface of the cyst ended in a thin tract in close contact to the pterygoid muscles. The most distal visible part of this tract was doubly ligated and transected in order to remove the parapharyngeal portion of the lesion. (Fig. 2a).

#### Endaural approach

A tympanomeatal flap was then elevated up to the level of the annulus, allowing the dissection of the cutaneous lining of the abnormal bony tunnel, originating from the inferior portion of the external auditory canal in close proximity to the annulus. Excision of about 1 cm of the cutaneous lining of the abnormal bony tunnel was then realized (Fig. 2b). A canaloplasty was performed by harvesting tragal cartilage and inserting it at the level of the opening of the bony tunnel. Biologic glue was applied over the cartilage as a seal.

#### Parotidectomy approach

A parotidectomy approach was then realized under NIM2 monitoring of the facial nerve. Excision of the inferior portion of the deep parotid lobe was carried out resulting in identification of the continuation of the previously described bony tunnel. The tunnel was located 1 cm deep (medial) to the facial nerve. Under the microscope, the tunnel was opened along the ¾ of its circumference using a burr drill in order to reveal the fistulous tract. The tract was followed until its visible connection with the previous ligation of the deep tract of the parapharyngeal cyst, thus confirming that our patient presented with a single lesion, which was unclear on preoperative MRI.

### Post-operative care and follow-up

**Fig. 3:**
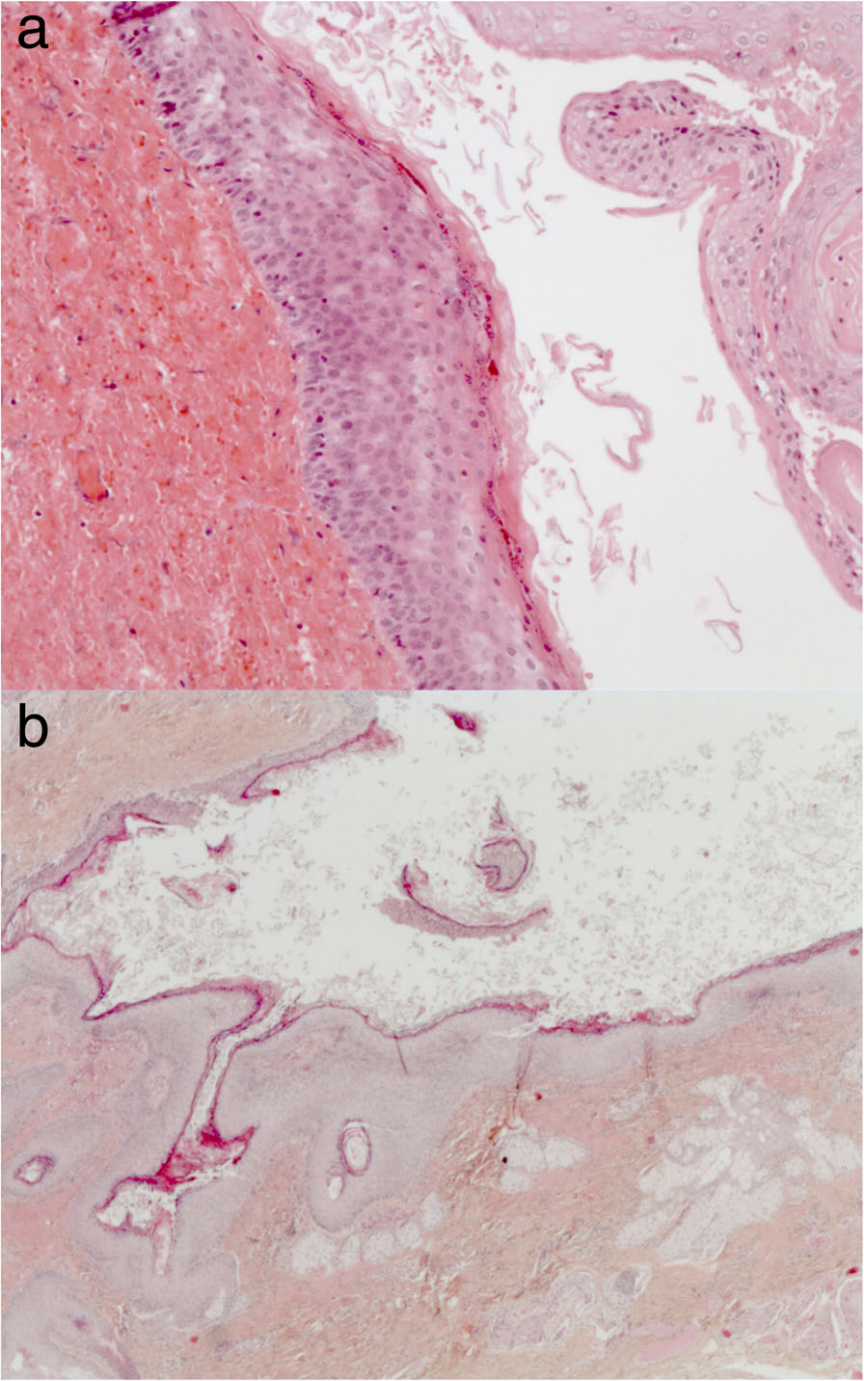
Histopathological analysis. **a**. High power H&E slide of the pharyngeal cyst revealing squamous epithelium, intraluminal keratin debris and the absence of pilosebaceous units, respiratory epithelium or associated lymphoid tissue. **b**. Low power H&E slide of the external auditory canal lesion revealing keratinized squamous epithelium with abundant intraluminal keratin debris

The patient’s post-operative course was uncomplicated. He was placed on a 7-day course of amoxicillin/clavulanic acid. The neck drain was removed on the second post-operative day. He was then discharged on the third post-operative day. Final pathology of the tissue excised from both the ear and parapharyngeal regions was consistent with the diagnosis of a first branchial cleft cyst, revealing cystic spaces filled with keratin debris and lined by stratified squamous epithelium containing pilosebaceous units (Fig. 3a and b). The patient was last seen over 1 year post-operatively; he remains asymptomatic with no clinical evidence of residual or recurrent disease and no facial paralysis. An audiogram was performed and revealed normal hearing.

## Discussion

To our knowledge, our patient represents the first case of a first branchial cyst with lower and medial extension to the parapharyngeal space at the level of the oropharynx. Such an extension is usually reserved for second branchial cleft cysts, and even in this case remains a rare occurrence [3]. Embryologically, this can be explained by the fact that the first branchial apparatus gives rise to pharyngeal and oral structures, in particular to the Eustachian tube and to the anterior two-thirds of the tongue. One may wonder whether our case represents 1. a true pharyngeal extension of a first branchial cleft cyst, 2. the simultaneous occurrence of a first and second branchial cleft cyst, which has previously been reported [4], or, possibly, 3. a “hybrid” first and second branchial anomaly. Such hybrid cases remain extremely rare in the literature with only a couple reported to date [5, 6]. Histopathological analysis of our case is in favor of a first branchial cleft cyst given the absence of both respiratory epithelium and associated lymphoid tissue, two common findings in second branchial cleft cysts [7]. As well, the sequential dissection of both the superior then the inferior portions of the lesion by various approaches (transoral and transcervical) during the operation seemed in complete continuity, further supporting the diagnosis of an atypical first branchial cleft cyst and eliminating the possibility of the simultaneous occurrence of first and second branchial cleft cysts. Lastly, the possibility of a “hybrid” first and second branchial anomaly should also be considered, although less likely given the lack of respiratory epithelium or lymphoid tissue.

Clinically, other cases of malformations of the first branchial apparatus involving pharyngeal and parapharyngeal structures have been reported. To date, at least three cases of medial parapharyngeal extension of first branchial cleft cysts at the level of the nasopharynx through the Eustachian tube have been published [8, 9]. Do et al. reported a complex fistulous anomaly of the first branchial apparatus encompassing parapharyngeal and pharyngeal lesions at the level of both the naso- and the oropharynx [10]. Vaughan et al. [11] described the association of a first branchial cleft sinus with a hairy polyp implanted on the posterior aspect of the soft palate.

From a practical point of view, our case highlights the importance of always considering a branchial cleft anomaly, either first or second, as the underlying etiology for any symptomatology combining otologic and cervical or pharyngeal locations. Both the general and the pediatric Otolaryngologist-Head and Neck Surgeon should bear this in mind. Proper treatment of branchial cleft cysts involves complete surgical excision of both the lesion and any associated fistulous tract, given the high rate of recurrence if any tissue is left behind [12–14]. Our three-phase surgical approach successfully accomplished this, despite the technical complexity of operating around a bony canal while preserving the facial nerve. In terms of management decision-making, the main constraint was the parapharyngeal extension. Given our high index of suspicion of a branchial cleft cyst, complete removal was essential. We considered classical approaches to the parapharyngeal space such as mandibular swing, but we were worried about morbidity, especially in a child. Furthermore, we were concerned that a pure transcervical approach would not give us enough exposure to properly and fully excise the lesion. The role of the surgical robot in our case was therefore to achieve complete excision while avoiding undue morbidity.

The surgical robot is emerging in the literature as a novel tool for the treatment of pediatric pathologies, in particular for general surgery cases [15]. Recently, its application has expanded to pediatric otolaryngology to include lingual tonsillectomy and airway surgery [16–18]. Our case represents a very interesting application of the surgical robot, without which a mandibulotomy would likely have been necessary for transoral access and visualization, especially in the context of a pediatric patient with an intrinsically smaller anatomy and limited mouth opening. We believe that, in our particular case, combining classical and robotized surgical approaches was very helpful in achieving complete surgical excision.

## Conclusion

In conclusion, we hereby describe an unusual presentation of a first branchial cleft cyst with a pharyngeal extension, possibly a hybrid first and second branchial cyst. Otolaryngologists should keep this possibility in mind when evaluating a child with both otologic and neck symptoms. Thorough pre-operative radiologic workup should aim at establishing the presence of a connecting fistulous tract. Operative management is greatly aided by combining traditional and robotic approaches.

## Data Availability

Not applicable.
